# Thiazolidinedione Use Is Associated with a Borderline Lower Risk of Multiple Myeloma and a Significantly Lower Risk of Death in Patients with Type 2 Diabetes Mellitus in Taiwan

**DOI:** 10.3390/cancers15174276

**Published:** 2023-08-26

**Authors:** Chin-Hsiao Tseng

**Affiliations:** 1 Department of Internal Medicine, National Taiwan University College of Medicine, Taipei 10051, Taiwan; ccktsh@ms6.hinet.net; 2 Division of Endocrinology and Metabolism, Department of Internal Medicine, National Taiwan University Hospital, Taipei 10002, Taiwan; 3 National Institute of Environmental Health Sciences of the National Health Research Institutes, Zhunan 35053, Taiwan

**Keywords:** multiple myeloma, pharmacoepidemiological study, pioglitazone, rosiglitazone, thiazolidinedione

## Abstract

**Simple Summary:**

The effects of thiazolidinedione (TZD) on multiple myeloma have not been investigated in humans. The reimbursement database of Taiwan’s National Health Insurance was used to select a propensity score-matched 86,999 pairs of TZD users and non-users who had a new-onset type 2 diabetes mellitus from 1999 to 2005. They were followed up from 1 January 2006 until 31 December 2011. The findings suggest a 35% lower risk of multiple myeloma associated with TZD with borderline significance. Subgroup analyses suggest a null association in patients aged <65 years but a significantly 45% lower risk in patients aged ≥65 years. Patients who have been treated with TZD may have a survival advantage.

**Abstract:**

Background: Thiazolidinedione (TZD) exerts anti-proliferative effects on multiple myeloma (MM) cells. However, there has not been any human study investigating the risk of MM associated with TZD use. Methods: We used Taiwan’s National Health Insurance database to identify 423,949 patients who had been newly diagnosed with diabetes mellitus between 1999 and 2005. After excluding ineligible patients, 86,999 pairs of patients with and without the use of TZD (rosiglitazone or pioglitazone) that had been matched based on propensity score were selected for a follow-up for MM until 31 December 2011. The hazard ratios for MM were estimated using Cox regression and weighted using a propensity score. Results: After a median follow-up of 4.6 years and 4.7 years in ever users and never users of TZD, 32 and 47 cases were diagnosed with MM, respectively. A 35% lower risk (though not statistically significant) was observed among ever users (hazard ratio 0.652, 95% confidence interval: 0.416–1.023, *p* = 0.0625). When ever users were divided by the median (15 months) cumulative duration of TZD therapy, the hazard ratios (95% confidence interval) for the lower and upper medians were 0.706 (0.394–1.264) and 0.603 (0.346–1.051), respectively. When treated as a continuous variable, the hazard ratio for every 1-month increment of the cumulative duration was 0.980 (95% confidence interval: 0.963–0.997, *p* = 0.0185). In the age subgroup analysis, a significantly lower risk could be seen in the older age subgroup of ≥65 years (hazard ratio 0.550, 95% confidence interval: 0.305–0.992, *p* = 0.0468). Additional analyses suggested that there were no interactions between TZD and some medications and between TZD and some clinical diagnoses, and that the use of TZD as a preventive drug for MM might not be cost-effective because a number-needed-to-treat of 5800 was too large. Survival analyses suggested that ever users had a significantly lower risk of death when all patients were analyzed (hazard ratio: 0.84, 95% confidence interval: 0.81–0.87, *p* < 0.0001 via a log-rank test) or when patients who developed MM were analyzed (hazard ratio: 0.40, 95% confidence interval: 0.19–0.86, *p* = 0.0153 via a log-rank test). Conclusions: In Taiwanese patients with type 2 diabetes mellitus, TZD use is associated with a borderline lower risk of MM, which is more remarkable in patients aged ≥65 years. Because of the low incidence of MM, the use of TZD for the prevention of MM may not be cost-effective. Patients who have been treated with TZD may have a survival advantage. Future research is required to confirm the findings.

## 1. Introduction

Multiple myeloma (MM) is a hematological malignancy characterized by bone marrow plasmacytosis with an increased production of immunoglobulins. Approximately 10% of all hematological cancers are MM and it represents around 1% of all cancers [1]. MM is the second most common hematological malignancy after lymphoma [2] and the most common clinical manifestations are hypercalcemia, renal failure, anemia and lytic bone lesions [1]. MM can be associated with some gene mutations, and epidemiological studies have shown its links with aging, male sex, obesity, diabetes mellitus and metabolic syndrome [1,3,4,5,6,7,8,9]. Dioxin exposure [10,11] and ionizing radiation [12] can also be risk factors but smoking is not [13]. MM may develop from an asymptomatic premalignant stage known as monoclonal gammopathy of undetermined significance, and a more advanced premalignant stage known as smoldering MM [1,2,14,15,16,17,18]. Firefighters exposed to the ashes during the terrorist attack on the World Trade Center in New York, USA, on 11 September 2001 suffered from a significantly higher risk of myeloma precursor disease [14]. The median age of MM diagnosis is 69 years in the USA and African Americans have a two-fold higher risk of MM than European Americans [2].

In Taiwan, an increasing secular trend in MM incidence has been observed from the late 1970s to the early 2010s [19,20]. The average age of MM incidence was 67.6 years [20] and the main increase in MM incidence occurred in the population aged ≥60 years [21]. At the time of MM diagnosis, a high proportion of the patients had clinical manifestations such as anemia (35.3%), bone fracture (18.0%), pneumonia (17.3%) and renal disease (16.4%) [20]. The independent risk factors associated with hospital mortality in patients with MM include hemodialysis, male sex, old age and catastrophic illness [22]. In the analysis of an Asian cohort consortium, obesity was a significant risk factor of MM mortality but smoking and alcohol drinking were not [23]. Pre-existing diabetes may have an adverse effect on the survival of the MM patients [24] and an early mortality within 60 days of an MM diagnosis was as high as 12.6% in a case series study conducted in Taiwan [25].

Thiazolidinedione (TZD) is a class of oral antidiabetic drug that improves insulin resistance by acting as an agonist to the nuclear hormone receptor of peroxisome proliferator-activated receptors gamma (PPARγ) [26,27,28], and PPARγ activation may affect the risk of cancer [29,30]. Human MM cells express PPARγ, and an exposure to PPARγ ligands [31,32] or an overexpression of PPARγ [33] may induce apoptosis and suppress the growth of MM cells. Previous in vitro studies have shown that TZDs including troglitazone, rosiglitazone and pioglitazone may exert such a potential benefit [34,35,36,37]. Furthermore, TZD can rapidly activate adenosine monophosphate-activated protein kinase (AMPK) [38,39], which may also inhibit the growth of MM cells [40].

In Taiwan, only rosiglitazone and pioglitazone have ever been marketed in the class of TZD [41]. As we know, there is no previous human study that investigates the risk of MM associated with TZD use or investigates whether TZD might affect the survival of patients with T2DM. In this study, we used Taiwan’s nationwide database of National Health Insurance (NHI) to examine the possible effects of TZD.

## 2. Materials and Methods

To improve the equity of healthcare for all people, Taiwan has implemented a unique and compulsory healthcare system, the so-called NHI, since 1 March 1995. The NHI database has been described in more detail elsewhere [42,43,44]. The government can release the NHI database for academic research if the proposal has been approved after an ethics review. 

During the study period, disease diagnoses in the database were coded using the International Classification of Diseases, Ninth Revision, Clinical Modification (ICD-9-CM). According to this coding system, diabetes mellitus was coded 250.XX and MM was coded 203.0.

**Figure 1:**
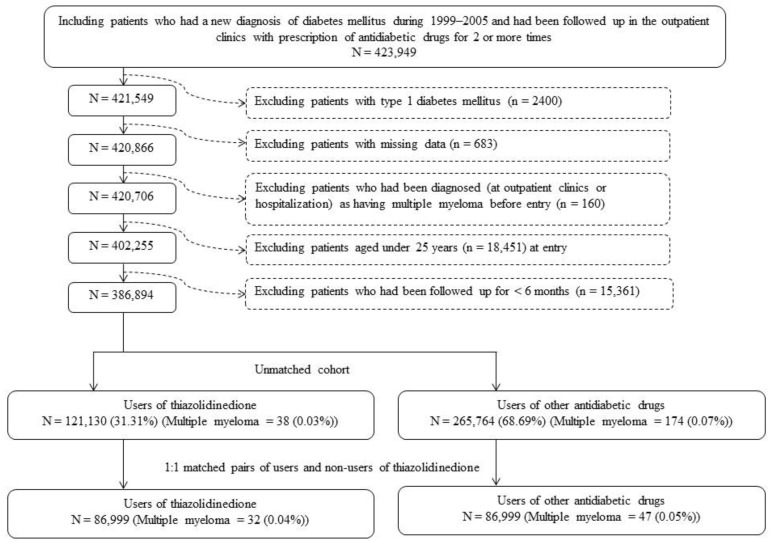
Procedures followed in selecting a propensity score-matched cohort of ever users and never users of thiazolidinedione derived from the reimbursement database of Taiwan’s National Health Insurance.

Logistic regression was used to create a propensity score (PS) from the independent variables which included the date of entry and all baseline characteristics listed in Table 1. We tried to enroll a cohort of ever users and never users with comparable characteristics based on the matching of their PSs. Figure 1 shows the procedures that had been followed. At first, we identified 423,949 patients who had been diagnosed with new-onset diabetes mellitus between 1999 and 2005 and had received prescriptions of antidiabetic drugs at outpatient clinics at least twice. Ineligible patients were then excluded, and we identified 386,894 patients as the unmatched cohort. A cohort consisting of 1:1 matched pairs of ever and never users was then created based on their PSs by using the Greedy 8 → 1 digit match algorithm [45]. As a result, 86,999 PS-matched pairs of ever users and never users of TZD were created for analysis.

Potential confounders used to create the PSs are listed in Table 1. The ICD-9-CM codes for the diagnoses can be seen elsewhere [42,43,44]. Helicobacter pylori (HP) infection was defined by a diagnosis (ICD-9-CM code 041.86) or a history of receiving an HP eradication therapy as detailed elsewhere [46]. Moderate to substantial agreements were observed between the claim data and medical records, with Kappa values ranging from 0.55 to 0.86 [47].

Standardized difference for each covariate was calculated as a test of balance diagnostics and a cutoff value of >10% was used to indicate a potential confounding [48].

The cumulative duration (months) of TZD use was calculated and its median cutoff was used to divide the exposure group into two subgroups. The incidence density of MM was calculated with regards to TZD exposure, i.e., for never users, ever users, and ever users divided by the median of the cumulative duration. The numerator of the incidence density was the incident case number of MM diagnoses by the end of the follow-up period. The denominator of the incidence density was the person-years of follow-up. The follow-up periods started on 1 January 2006 and ended on dates leading up to 31 December 2011, when any of the following events occurred first: a patient died, a new diagnosis of MM was given, or the last record in the database was reached.

Cox regression, incorporated with the inverse probability of treatment weighting using the PS, was used to estimate the hazard ratios. In the main analyses, we estimated the overall hazard ratios for ever users versus never users. A potential dose–response relationship was then assessed using the hazard ratios for the lower and upper medians of the cumulative duration versus never users, and by treating the cumulative duration as a continuous variable.

To examine whether the hazard ratio would be significant for either pioglitazone or rosiglitazone, analyses were conducted by excluding ever users of rosiglitazone and pioglitazone. Because age is an important risk factor for MM [2], we further estimated the hazard ratios in the younger (<65 years) and older (≥65 years) age subgroups.

Knowledge of the absolute risk reduction (ARR) and the number-needed-to-treat (NNT) are important for assessing the cost-effectiveness of a treatment [49]. We therefore calculated the ARR from the difference in incidence rates between never and ever users and the NNT from the reciprocal of the ARR [49].

The interactions between TZD and some drugs (i.e., the use of immunosuppressants, insulin, sulfonylurea, metformin, meglitinide, acarbose, aspirin, statin, fibrate, angiotensin converting enzyme inhibitors/angiotensin receptor blockers and calcium channel blockers) and medical conditions including Ebstein-Barr virus infection, human immunodeficiency virus disease, autoimmune disease and/or organ transplantation were investigated. The *p* values of the interaction terms were estimated using Cox regression and included the independent variables of TZD, all covariates in Table 1, and the interaction terms of TZD and each of the investigated variables, one at a time.

The Kaplan–Meier curves of survival-free probability were plotted for ever users and never users in all patients, and in patients who developed MM, respectively. The differences in survival between ever users and never users were tested using a log-rank test.

The SAS statistical software (version 9.4) developed by the SAS Institute (Cary, NC, USA) was used to perform statistical analyses. A *p*-value < 0.05 indicated statistical significance.

## 3. Results

Table 1 compares the baseline characteristics between never users and ever users selected from the NHI database based on PS matching. All covariates were well-balanced between the two groups because all values of standardized difference were <10%.

The median follow-up duration was 4.7 years for never users and 4.6 years for ever users. At the end of the follow-up period, 47 never-user patients were diagnosed with MM and 32 ever-user patients were diagnosed with MM. The median time span from the first use of TZD until the occurrence of MM was 50.33 months (range: 10.73–87.93 months) in the 32 cases of MM among ever users.

Table 2 shows the incidence of MM, and the overall hazard ratio in the main analysis as well as in the investigation of a possible dose–response relationship. The incidence rates of MM for ever users and never users were 8.70 per 100,000 person-years and 13.12 per 100,000 person-years, respectively. Although not statistically significant, the overall hazard ratio indicated a 35% lower risk of MM among ever users (hazard ratio: 0.652, 95% confidence interval: 0.416–1.023, *p* = 0.0625). In the analysis that divided the cumulative duration by the median, the hazard ratios indicated an even lower risk (though not significant) among those with a higher cumulative duration of exposure of ≥15.5 months. However, the hazard ratio of 0.980 reached statistical significance (95% confidence interval: 0.963–0.997, *p* = 0.0185) when the cumulative duration was analyzed as a continuous variable.

Table 3 shows the hazard ratios in subgroup analyses after excluding ever users of rosiglitazone (Model 1, comparing ever users of pioglitazone versus never users) and pioglitazone (Model 2, comparing ever users of rosiglitazone versus never users), respectively. Though not statistically significant, a lower risk associated with the use of pioglitazone (33%, Model 1) or rosiglitazone (17%, Model 2) could be seen. While dividing the patients into a younger age group (<65 years, Model 3) and an older age group (≥65 years, Model 4), TZD was significantly associated with a 45% lower risk in the older age group.

The calculated ARR was 0.017% (47/86,999 − 32/86,999) and the NNT was 5800. There was a lack of interaction between TZD and the assessed medications, and clinical diagnoses (data not shown).

**Figure 2:**
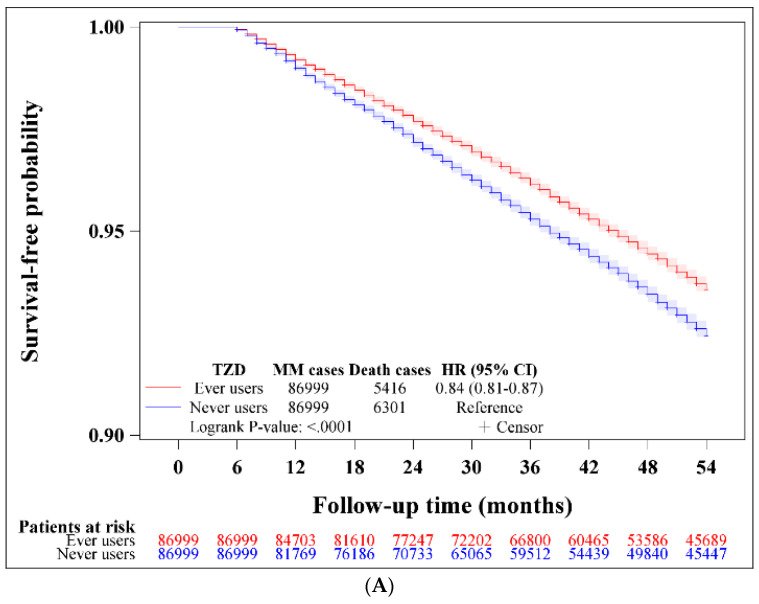

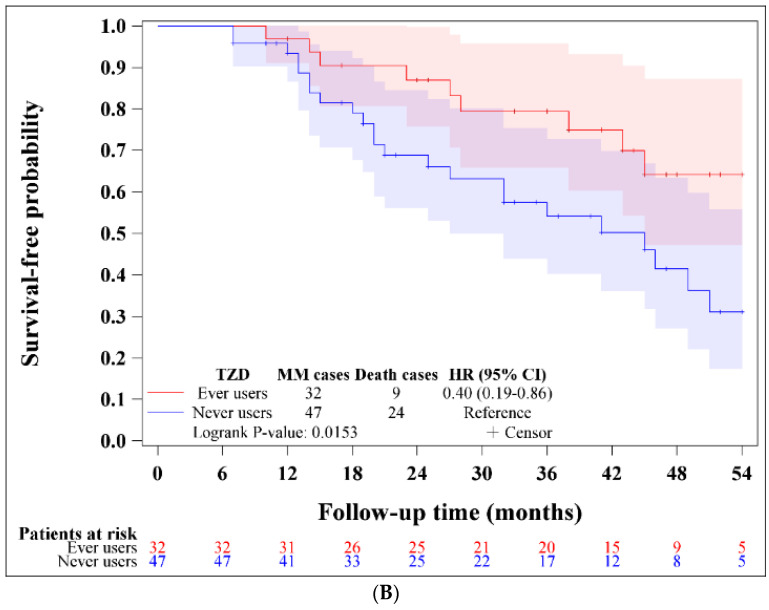
Survival-free probability in ever users and never users of thiazolidinedione (TZD) in all patients (**A**) and in patients who developed multiple myeloma (**B**). HR: hazard ratio, CI: confidence interval.

Figure 2 shows the Kaplan–Meier curves of the survival-free probability in ever users and never users of TZD in all patients (Figure 2A), and in the 32 ever users and 47 never users of TZD who developed MM (Figure 2B). The findings suggested a survival advantage in ever users of TZD compared to never users.

## 4. Discussion

### 4.1. Main Findings

This is the first study reporting a potential benefit of TZD on MM in patients with T2DM. Although not statistically significant, we found a 35% lower risk of MM among ever users of TZD in the main analysis (Table 2). In subgroup analyses, the beneficial effect was significant in patients aged ≥65 years. Although the benefit of TZD on MM risk was borderline significant (Table 2), patients who had been treated with TZD might have a significantly lower risk of death (Figure 2).

### 4.2. Explanations for an Attenuated Effect of TZD in Humans

Although earlier in vitro studies indicated a potential usefulness of TZD for the treatment of MM through mechanisms of suppressing angiogenesis [34], inactivating STAT3 [35], inhibiting the adhesive interactions of MM cells with bone marrow stromal cells [36] and acting as a mitochondrial inhibitor [38] and as an activator of AMPK [39,40], our study did not fully support a significant overall benefit in humans (Table 2). This could partly be explained by the low incidence of MM in our population, the lack of sufficient statistical power and the possible benefits only being observed in the elderly patients (Table 3). However, the benefit of TZD on MM could also be attenuated in humans because of the following reasons.

Obesity is an important risk factor for MM [5,7,8] and diabetes mellitus [50]. TZD use is associated with an increase in body weight [51], which might increase the risk of MM [5,7,8] and counteract the benefits of TZD that were demonstrated in preclinical studies [34,35,36,37,38,39,40]. Therefore, the benefit of TZD on MM would not be as remarkable in studies conducted on human beings (Table 2 and Table 3).

Recent studies have shown a complex cross-talk between bone marrow adipocytes and myeloma cells [52,53]. TZD may increase bone marrow adipocytes, resulting in an increased production of adipokines that may lead to bone loss and an increased potential for the bone metastasis of cancers like prostate cancer, breast cancer, lung cancer and MM [53]. Therefore, this would also exert an adverse effect on the development of MM.

It is also possible that the dosage used in the treatment of hyperglycemia in patients with T2DM might not be sufficient to exert an anti-cancer effect on MM.

### 4.3. Implications

This observational study has some clinical implications. First, because the benefit of TZD could only be significantly observed in patients aged ≥65 years (Table 3), and bladder cancer, a cancer that is age-related, has been warned as being a risk of pioglitazone use [54], the use of TZD among elderly patients should be balanced by a potential risk of bladder cancer. However, we did not show an increased risk of bladder cancer associated with either pioglitazone [55] or rosiglitazone [56]. On the other hand, a potential benefit of pioglitazone on prostate cancer, another age-related cancer in men, has been demonstrated in several recent studies [57,58] (though not consistently observed by others [59,60]). Currently, only pioglitazone in the class of TZD is available for the treatment of T2DM because troglitazone has never been approved in Taiwan and rosiglitazone has been withdrawn from the market since the warning of the potential risk of cardiovascular disease associated with its use [61]. Therefore, the clinical use of pioglitazone and its potential benefits on dementia [62,63], lipid profiles [51,64,65], non-alcoholic fatty liver disease [66,67,68], cardiovascular disease [69,70] and prostate cancer [57,58] should be more extensively explored to balance its beneficial and harmful effects [71].

Second, because TZD exerts an antimicrobial effect [27,28] and infection is the most common cause of early mortality in MM cases [25], it is worthwhile to investigate the potential usefulness of TZD as an adjuvant therapy for patients with early MM. PPARγ agonists may potentiate the cytotoxic effect of valproic acid in MM cells [72], and early in vitro studies suggested that all-trans retinoic acid can potentiate the anti-MM cell effect of rosiglitazone [73]. Furthermore, the PPARγ ligand RWJ-241947 (MCC-555) may enhance the apoptosis of MM cells induced by arsenic trioxide [74]; therefore, it is worthwhile to further investigate the potential usefulness of a combination use of TZD and existing chemotherapeutic agents for the treatment of MM. Although troglitazone has been withdrawn from the market, it is worthwhile to investigate whether troglitazone can be repurposed as a therapeutic agent for MM. However, the potential adverse effect of an increase in body weight associated with TZD, as well as the hepatotoxicity associated with troglitazone, should also be taken into account.

Third, some novel TZDs without adipogenic and osteolytic effects are being developed [75,76] and some are being investigated for their usefulness in the treatment of MM [77,78,79]. Furthermore, it is interesting that an early study showed that PPARγ antagonists (GW9662 and T0070907) exhibited more potent antiproliferative effects on MM cells than the PPARγ agonist pioglitazone, and that a combination of PPARγ antagonists and pioglitazone might exert a greater inhibitory effect on the growth of MM cells [80]. Therefore, the usefulness of novel TZDs, as well as a potential utility of various combinations of PPARγ antagonists and agonists in the prevention and treatment of MM, is awaiting future investigation.

Fourth, patients with MM that are treated with novel therapeutic agents such as thalidomide, bortezomib and lenalidomide may have an improved survival rate, which might lead to a new concern with the long-term risk of second primary malignancies [81,82,83]. However, this was not supported by a recent study conducted in Taiwan that showed, after controlling for potential confounders, a significantly lower incidence of second primary malignancies in MM patients who had been treated with these novel agents than patients treated with chemotherapy alone [84]. Whether a combination of TZD and these novel therapeutic agents may exert a greater improvement in the survival of MM patients, and an even lower incidence of second primary malignancies, is important clinical research.

Fifth, because of the very low incidence of MM in Taiwanese people with T2DM (Table 2), the calculated ARR of 0.017% was too small and the NNT of 5800 was too large. Therefore, it might not be cost-effective to use TZD for the prevention of MM in a large population. Additionally, the use of TZD is associated with a wide range of potential side effects including weight gain, fluid retention, congestive heart failure, macular edema, bone loss and fractures, and an increased risk of myocardial infarction with rosiglitazone and bladder cancer with pioglitazone [85]. These potential side effects of TZD also do not favor the use of TZD as a preventive agent for MM in individuals who suffer from side effects. Although the NHI does not restrict the use of TZD (only pioglitazone is now available) in current reimbursement guidelines, the local Diabetes Association in Taiwan recommends TZD as a second- or third-line therapy for hyperglycemia in patients with T2DM in the absence of potential side effects. This might also restrict the potential usefulness of TZD for the prevention of MM from a practical point of view. However, the significantly lower risk of death in patients treated with TZD (Figure 2) provides a hint toward the possible utility of TZD as an adjuvant therapy for MM.

### 4.4. Strengths

By using a nationwide database that covers > 99% of the population, the findings can be more readily generalized to the whole population. This study can also be free from self-reporting bias because we used existing medical records. Different socioeconomic status may lead to a detection bias and can be a serious problem in some studies. However, for the following reasons, this was unlikely in the present study: (1) in the NHI healthcare system in Taiwan, cancer is considered as a catastrophic illness, and those who have a certified diagnosis of cancer can be waived of most medical copayments; and (2) much of the cost-sharing for patients who use the NHI service can be waived if the patients have low income, if they are veterans, or if they are getting drug refills for chronic disease.

### 4.5. Limitations

This study may have some limitations. First, the NHI database does not collect information on some potential risk factors of MM. These may include radiation exposure, body height, body weight and genetic parameters. Although diagnoses of ocular pterygium, used as a surrogate marker for UV sunlight exposure, were balanced between ever and never users of TZD (Table 1), we could not completely exclude the possible confounding effect of radiation exposure from various sources. We did not have anthropometric data such as body height and body weight and could only use diagnoses of obesity for analyses. Although diagnoses of obesity were balanced between ever and never users (Table 1), we believed that only patients with morbid or severe obesity would be labelled with such a diagnosis in the database. Although genetic parameters were not available, we believed that their effects would be minimal if they were not related to the exposure of TZD. If the distributions of other unidentified risk factors were not differential, they would probably only lead to a bias toward the null for the estimated hazard ratios [86].

Second, some Chinese herb drugs [87] as well as vitamin D deficiency [88] may affect the risk of MM. We did not have sufficient information to investigate whether the use of Chinese herb drugs or other supplements might have played important confounding roles in the study.

Third, we did not have the pathology of the bone biopsies for a confirmation of the diagnoses and for additional analyses.

Fourth, because this is the first study reporting a potential role of TZD in the prevention of MM in an Asian ethnicity, and because of a possible lack of sufficient statistical power due to the small case numbers of MM and the limited follow-up time, the findings should be further confirmed through future research on other ethnicities.

Fifth, some novel antidiabetic drugs such as incretin mimetics/enhancers and sodium-glucose cotransporter 2 inhibitors have been marketed after the enrollment period. These novel drugs may also affect the risk of cancer [89,90,91,92,93,94] and their use during the follow-up period might have played confounding roles in this study. The first to be marketed among them was sitagliptin, which was approved in Taiwan in July 2007 [89]. In secondary analyses, the results and conclusions of this study were not affected when patients who happened to use these novel drugs during follow-up were excluded (data not shown).

## 5. Conclusions

Although not statistically significant, this is the first human study that suggests a 35% lower risk of MM among patients with T2DM who have ever used TZD. This benefit would be more remarkable (45% risk reduction) and significant in elderly patients aged ≥65 years. There seems to be a lack of interaction between TZD and some drugs and between TZD and some clinical diagnoses regarding its role in MM development. The use of TZD as a preventive agent for MM may not be cost-effective because of the large NNT. However, the survival benefit in patients who had been treated with TZD suggests a future direction for the investigation on the positioning of TZD in the treatment of diabetes and MM. Our findings should be deemed as preliminary and additional research using existing large datasets, or through the design of clinical trials, is required.

## Figures and Tables

**Table 1:** Characteristics of propensity score-matched cohort of ever and never users of thiazolidinedione.

Characteristics	Ever Users (*n* = 86,999)		Never Users (*n* = 86,999)		Standardized Difference
	*n*	%	*n*	%	
**Basic data**					
Age * (years)	59.32	12.3	59.08	11.72	−1.85
Diabetes duration * (years)	5.57	2.48	5.53	5.53	−2.55
Sex (men)	45,907	52.77	45,835	52.68	−0.07
Occupation					
I	34,333	39.46	34,394	39.53	
II	18,635	21.42	18,864	21.68	0.62
III	17,510	20.13	17,605	20.24	0.39
IV	16,521	18.99	16,136	18.55	−1.13
Living region					
Taipei	31,203	35.87	31,090	35.74	
Northern	9601	11.04	9513	10.93	−0.34
Central	16,908	19.43	16,981	19.52	0.29
Southern	12,060	13.86	12,151	13.97	0.23
Kao-Ping and Eastern	17,227	19.8	17,264	19.84	0.21
**Major comorbidities of diabetes mellitus**					
Obesity	4011	4.61	3977	4.57	−0.29
Hypertension	64,495	74.13	64,187	73.78	−0.72
Dyslipidemia	63,152	72.59	63,362	72.83	0.55
**Major complications of diabetes mellitus**					
Hypoglycemia	1367	1.57	1351	1.55	−0.01
Eye diseases	15,892	18.27	15,562	17.89	−0.70
Nephropathy	17,747	20.4	17,747	20.4	−0.95
Diabetic polyneuropathy	17,523	20.14	17,214	19.79	−0.55
Peripheral arterial disease	16,722	19.22	16,538	19.01	−0.42
Stroke	20,546	23.62	20,012	23	−1.37
Ischemic heart disease	34,784	39.98	34,287	39.41	−1.14
**Comorbidities/clinical symptoms that might affect exposure or outcome**					
Tobacco abuse	1948	2.24	1930	2.22	−0.10
Alcohol-related diagnoses	4676	5.37	4699	5.4	0.11
Chronic obstructive pulmonary disease	37,410	43	36,851	42.36	−1.31
Valvular heart disease	6855	7.88	6715	7.72	−0.67
Heart failure	12,187	14.01	11,822	13.59	−1.18
Head injury	1228	1.41	1190	1.37	−0.41
Dementia	4238	4.87	4014	4.61	−1.21
Parkinson’s disease	1694	1.95	1609	1.85	−0.69
Helicobacter pylori infection	641	0.74	628	0.72	−0.20
Hepatitis B virus infection	602	0.69	1722	1.98	−0.07
Hepatitis C virus infection	3192	3.67	3172	3.65	−0.10
Cirrhosis of liver without mention of alcohol	3361	3.86	3370	3.87	0.07
Other chronic nonalcoholic liver disease	8434	9.69	8472	9.74	0.12
Epstein-Barr virus infection	602	0.69	605	0.7	0.05
Human immunodeficiency virus disease	53	0.06	41	0.05	−0.65
Autoimmune diseases	6815	7.83	6734	7.74	−0.35
Organ transplantation	234	0.27	224	0.26	−0.20
Immunosuppressants	3099	3.56	3014	3.46	−0.52
Insomnia	21,631	24.86	21,228	24.4	−1.07
Malaise and fatigue	4411	5.07	4301	4.94	−0.63
History of some disorders of the central nervous system	18,264	20.99	18,013	20.7	−0.70
Ocular pterygium	3621	4.16	3661	4.21	0.25
Disorders of thyroid gland	9010	10.36	8876	10.2	−0.66
Nutritional deficiencies	2194	2.52	2150	2.47	−0.30
Depression	5953	6.84	5865	6.74	−0.43
Bone fractures	17,997	20.69	17,693	20.34	−0.87
Benign neoplasm of bone and articular cartilage	385	0.44	340	0.39	−0.85
**Antidiabetic drugs**					
Insulin	2751	3.16	2697	3.1	−0.63
Sulfonylurea	56,575	65.03	58,437	67.17	4.23
Metformin	56,695	65.17	56,695	65.17	0.92
Meglitinide	5205	5.98	5182	5.96	−0.19
Acarbose	7518	8.64	7466	8.58	−0.52
**Drugs commonly used by diabetes patients**					
Aspirin	45,717	52.55	45,375	52.16	−0.73
Statin	44,908	51.62	44,915	51.63	0.04
Fibrate	30,913	35.53	31,122	35.77	0.58
Angiotensin converting enzyme inhibitor/angiotensin receptor blocker	55,466	63.75	55,366	63.64	−0.10
Calcium channel blocker	46,946	53.96	46,636	53.61	−0.61

* Age and diabetes duration are shown in mean and standard deviation.

**Table 2:** Incidence rates of multiple myeloma and hazard ratios of thiazolidinedione exposure in the propensity score-matched cohort.

Thiazolidinedione Use	Incident Case Number	Cases Followed	Person-Years	Incidence Rate (per 100,000 Person-Years)	Hazard Ratio	95% Confidence Interval	*p* Value
Never users	47	86,999	358,126.16	13.12	1.000		
Ever users	32	86,999	368,026.76	8.70	0.652	(0.416–1.023)	0.0625
Median of cumulative duration of thiazolidinedione therapy (months)							
Never users	47	86,999	358,126.16	13.12	1.000		
<15.5	15	43,491	160,490.73	9.35	0.706	(0.394–1.264)	0.2417
≥15.5	17	43,508	207,536.04	8.19	0.603	(0.346–1.051)	0.0743
Cumulative duration of thiazolidinedione therapy treated as a continuous variable					0.980	(0.963–0.997)	0.0185

**Table 3:** Subgroup analyses.

Models	Incident Case Number	Cases Followed	Person-Years	Incidence Rate (per 100,000 Person-Years)	Hazard Ratio	95% Confidence Interval	*p* Value
1. Excluding ever users of rosiglitazone							
Never users	47	86,999	358,126.16	13.12	1.000		
Ever users	11	34,914	123,073.04	8.94	0.673	(0.348–1.300)	0.2380
2. Excluding ever users of pioglitazone							
Never users	47	86,999	358,126.16	13.12	1.000		
Ever users	10	20,109	89,453.24	11.18	0.832	(0.420–1.646)	0.5966
3. Age < 65 years							
Never users	15	58,267	247,605.60	6.06	1.000		
Ever users	15	59,680	262,621.53	5.71	0.927	(0.543–1.898)	0.8362
4. Age ≥ 65 years							
Never users	32	28,732	110,520.56	28.95	1.000		
Ever users	17	27,319	105,405.24	16.13	0.550	(0.305–0.992)	0.0468

## Data Availability

Public availability of the dataset is restricted by local regulations to protect privacy.
